# New Journals

**Published:** 1879-02

**Authors:** 


### New Journals.

*St. Louis Courier of Medicine* makes its first appearance with January of 1879. It has the personal appearance and moral worth of an established reality, and we wish it and its editors a signal success. Our old and much respected friend, Dr. A. J. Steele, graduate of the University of Buffalo, and House Physician and Surgeon to the Buffalo General Hospital, is the editor, with Drs. Hardway and Schauffeler as associates. Their first number is highly creditable to the editors and to the contributors who join them in their effort.

Detroit also sends *The Medical Advance*, to be devoted to the publication of short, illustrated articles in all departments of medicine and surgery, but largely to medicines, new and old.

